# Effects and mechanisms of resveratrol on the amelioration of oxidative stress and hepatic steatosis in KKAy mice

**DOI:** 10.1186/1743-7075-11-35

**Published:** 2014-08-12

**Authors:** Wei Zhu, Sifan Chen, Zilun Li, Xiaohong Zhao, Wenxue Li, Yanshuang Sun, Zili Zhang, Wenhua Ling, Xiang Feng

**Affiliations:** 1 School of Public Health, Sun Yat-Sen University, Guangzhou, Guangdong, People’s Republic of China; 2 Guangdong Provincial Key Laboratory of Food, Nutrition and Health, Guangzhou, Guangdong, People’s Republic of China; 3 Guangzhou Center for Disease Control and Prevention, Guangzhou, Guangdong, People’s Republic of China; 4 Division of Vascular Surgery, The First Affiliated Hospital, Sun Yat-sen University, Guangzhou, Guangdong, People’s Republic of China; 5 Department of Nephrology, The First Affiliated Hospital, Sun Yat-sen University, Guangzhou, Guangdong, People’s Republic of China

**Keywords:** NAFLD, Lipid metabolic disorder, Oxidative stress, Resveratrol, Sirt1, AMPK

## Abstract

**Background:**

The exact mechanism of the protective role of Resveratrol (Res) in lipid metabolism and oxidative stress is not well elucidated. The present study aimed to investigate the potential benefits and possible mechanisms of Res on the amelioration of oxidative stress and hepatic steatosis in a KKAy mouse model.

**Methods:**

A total of 30 KKAy male mice were randomly divided into three groups: a normal chow group, a low resveratrol group and a high resveratrol group. After a 12-wk study period, serum levels of TG, TC, LDL-C and HDL-C, the liver content of TG and TC, ROS, GSH, GPx, SOD and MDA levels were measured. Ectopic lipid deposition was observed in sectioned frozen liver tissues. The mRNA levels of ATGL and HSL in the liver tissues were determined via real-time PCR. Furthermore, the protein expression of p47phox, gp91phox, ATGL, HSL, Sirt1, AMPK and FOXO1 were analyzed using western blotting.

**Results:**

Following Res supplementation, serum levels of TG and MDA were decreased, while the HDL-C and SOD levels were increased in KKAy mice. Furthermore, Res treatment increased GSH and GPx in liver tissues, while it decreased ROS. In addition, Res significantly reduced hepatic steatosis. After Res treatment, concentrations of p47phox (membrane) and gp91phox proteins were reduced, while p-HSL, HSL and ATGL protein expression levels were increased. Mechanistically, the levels of Sirt1, p-AMPK and p-FOXO1 expression in the liver tissues were up-regulated following supplementation with Res, and FOXO1 protein was released from the nucleus into the cytoplasm.

**Conclusions:**

Res is able to attenuate hepatic steatosis and lipid metabolic disorder and enhance the antioxidant ability in KKAy mice, possibly by up-regulating Sirt1 expression and the phosphorylation of AMPK.

## Introduction

Non-alcoholic fatty liver disease (NAFLD) affects up to 15 to 20% of the general population and is the most common cause of chronic liver disease. NAFLD includes a variety of histological conditions ranging from simple steatosis to non-alcoholic steatohepatitis (NASH), liver fibrosis, cirrhosis, and hepatocellular carcinoma [1]. Although NAFLD is known to be correlated with many etiological factors, its pathogenesis remains poorly understood. The most common risk factors are obesity, insulin resistance, dyslipidemia, and genetic susceptibility, which affect hepatic triglyceride homeostasis [2]. Previous studies have confirmed that hepatic steatosis mostly results from the increased free fatty acid (FFA) supply due to increased lipolysis and/or increased intake of dietary fat, which contributes to 60% of hepatic fat content [3]. Hormone-sensitive lipase (HSL) and adipose triglyceride lipase (ATGL), two enzymes critical for lipolysis in adipose tissues, have been found to play an important role in hepatic lipid homeostasis [4]. Dysfunctional lipolysis affects energy homeostasis and may contribute to the pathogenesis of hepatic steatosis. Triglyceride accumulation in patients with NASH may eventually lead to the development of cirrhosis. However, to date, there are no therapies proven effective in halting NAFLD progression. Hence, it is of great clinical value to further understand the mechanisms underlying the progression of NAFLD and to identify novel therapeutic targets.

Resveratrol (Res) is known as a natural polyphenol, which is synthesized by plants and is present in the skins of grapes, nuts, and, in the highest concentration, red wine [5]. Previous studies have shown that Res may be a cardioprotective, cancer chemopreventive and chemotherapeutic agent [6]. Recently, Res has gained attention for its protection against metabolic disease. Baur JA et al. reported that Res improved health and survival in mice fed a high-calorie diet [7]. Additionally, Res can decrease liver lesions induced by alcohol and hepatotoxic drugs [8]. Due to its beneficial effects on energy metabolism, Res is suggested to be a promising new therapeutic approach for treating metabolic diseases.

Sirt1, an NAD-dependent deacetylase, is a key protein involved in many of the effects of Res. An in vitro study showed that Res, a Sirt1 activator, enhanced insulin sensitivity in a Sirt1-dependent manner and attenuated high-fat-diet-induced insulin resistance [9]. The cellular energy sensor, AMP-activated protein kinase (AMPK) has been demonstrated to be closely related to insulin resistance and hepatic steatosis, which could be of great relevance for the treatment of metabolic syndrome [10]. Recent studies have shown that AMPK activation increased the fatty aid oxidation of skeletal muscle in rats [11]. Additionally, AMPK is reported to regulate metabolism by modulating Sirt1 activity [12]. Additional studies have shown that the tumor suppressor LKB1 kinase could activate AMPK and regulate apoptosis in response to energy stress via the Sirt1 pathway [13].

The KKAy mouse was originally developed by crossing the KK mouse with the yellow obese mouse (Ay mouse), which are obese, hyperglycemic, hyperinsulinemic, and insulin resistant, therefore applied as animal model for the research on obesity or metabolic disorders [14]. The C57BL/6 J mice were chosen as the control group. So far, whether the metabolic protective effect of Res also occurs in KKAy mice remains unclear. Moreover, the exact mechanism of the protective role of Res is not elucidated and is the focus of our study. In a previous study, we discovered that Res could improve insulin sensitivity and ameliorate insulin resistance in KKAy mice, which may be associated with the up-regulation of Sirt1 protein in the liver and soleus muscles and, consequently, Sirt1 and AMPK activation [15]. In this study, we wanted to investigate the effect and mechanism of Res on the regulation of oxidative activation and lipid metabolism.

## Materials and methods

### Materials

Resveratrol was purchased from Yixin Pharm, Inc. (Zhejiang, China); The triglyceride (TG) kit, total cholesterol (TC) kit, low density lipoprotein cholesterol (LDL-C) kit, high density lipoprotein cholesterol (HDL-C) kit, superoxide dismutase (SOD) kit, malonaldehyde (MDA) kit, free fatty acid (FFA) kit, reactive oxygen species (ROS) kit, glutathione (GSH) kit, glutathione peroxidase (GPx) kit, and the coomassie brilliant blue kit were purchased from Jiancheng Bioengineering Institute (Nanjing, China); rabbit anti-mouse p47phox polyclonal antibody was purchased from LifeSpan Biosciences, Inc. (WA, USA); rabbit anti-mouse Sirt1 monoclonal antibody and the gp91phox polyclonal antibody were procured from Abcam, Inc. (Cambridge, UK); Adipose triglyceride lipase (ATGL), hormone-sensitive lipase (HSL), and p-HSL (Ser660) antibodies were purchased from Santa Cruz Biotech (Texas, USA); the primary antibodies including rabbit anti-mouse AMPK, p-AMPK (Thr172), FOXO1, and p-FOXO1 (Thr24) were purchased from Cell Signaling Technology (MA, USA); GAPDH antibody was obtained from Boster, Inc. (Wuhan, China); the BCA kit and HRP-labeled goat anti-rabbit secondary antibody were from Beyotime, Inc. (Jiangsu, China).

### Experimental animals

A total of 30 8-week-old KKAy male mice were obtained from Jackson Laboratory (Bar Harbor, ME). A total of 10 8-week-old C57BL/6 J male mice from the Animal Experimental Center of Guangdong Province (Guangdong, China) were chosen as the control group. All mice were maintained in a temperature- and humidity-controlled environment with a 12-h light/dark cycle and fed a standard laboratory chow for a 1-wk acclimation period. Subsequently, the KKAy male mice were randomly divided into 3 intervention groups based on body weight: a standard chow intake group (KKAy group), a low resveratrol group (KKAy + Low Res) and a high resveratrol group (KKAy + High Res). The control and KKAy groups were fed a standard AIN93G diet, while the Low Res and High Res groups were fed a standard AIN93G diet supplemented with Res at doses of 2 and 4 g/kg diet, respectively. The dosages of Res treatment were referred to the previous publication [16]. Blood samples of mice were drawn from the orbital veins for blood lipid measuring before the experimental intervention. All animals were allowed free access to food and water throughout the study. Body weight (BW) and food intake were measured weekly. After a 12-wk study period, mice of all groups were sacrificed under anesthesia after fasting for 12 h. Serum samples were collected and stored at -80°C prior to use. The liver tissues were removed, weighed and also stored at -80°C prior to use. This study was approved by the Institutional Animal Care and Use Committee at Sun Yat-sen University.

### Serum analyses

Serum levels of TG, TC, LDL-C and HDL-C were quantified using commercial enzyme-linked immunosorbent assay (ELISA) kits according to the manufacturer’s instructions.

### Liver tissue analyses

The hepatic index was obtained by dividing the wet liver weight by rat weight and multiplying by 100. Fresh liver tissue samples of 500 mg each were prepared, ground down in 0.5 mL saline solution, extracted with a mixture of chloroform and methanol, and then stored overnight at 4°C after vibration. The liver tissue homogenates were centrifuged at 3500 rpm for 20 min the next day. Next, the lower-phase liquid was collected to measure the TG and TC contents using specific kits according to the manufacturer’s instructions.

### Measurement of SOD, MDA, FFA, ROS, GSH, and GPx levels

The serum FFA, SOD and MDA contents were detected according to the manufacturer’s instructions. Furthermore, prepared fresh liver tissue samples were ground down in saline solution to make 10% liver tissue homogenates, followed by centrifuging at 3500 rpm for 20 min. Next, the supernatant was collected to measure the contents of SOD, MDA, ROS, GSH, and GPx using specific kits according to the manufacturer’s instructions. ROS measurement was referred to the previous described protocol [17].

### Frozen liver sections

In brief, fresh liver tissues were embedded in OCT buffer and frozen in liquid nitrogen. The frozen specimens were sectioned into 4 μm sections then stained with Oil Red O. The slides were then mounted with neutral gum and observed and photographed under the microscope.

### Real-time PCR analysis

The mRNA was extracted from the liver tissues using trizol and was used to synthesize cDNA. Specifically, 1 μL of 1 g/L RNA was mixed with 4 μL of 5 × Prime Script buffer, 1 μL RT enzyme mix I, 1 μL of oligo dT primer, 1 μL of random 6 mers, and 13 μL of DEPC water, and then incubated at 37°C for 15 min followed by 85°C for 5 s to inactivate reverse transcriptase. The cDNA was then used as the template to amplify the target genes using real-time PCR in a 20 μL system containing 2 μL of cDNA, 10 μL of 2 × SYBR Premix, 0.4 μL of Ex Taq, 0.4 μL of forward primer, 0.4 μL of reverse primer, 0.4 μL of 50 × ROX reference dye II, and 6.8 μL of dH2O under the following conditions: 95°C for 30 s, followed by 40 cycles at 95°C for 5 s, 55°C for 30 s, and 72°C for 30 s. The primers used for the ATGL amplification included the forward primer, 5-AACACCAGCATCCAGTTCAA-3 and reverse primer, 5-GGTTCAGTAGGCCATTCCTC-3; for HSL, the primers were: the forward primer, 5-GGCTCACAGTTACCATCTCACC-3, and the reverse primer, 5-GAGTACCTTGCTGTCCTGTCC-3. Primers used for beta-actin amplification were forward, 5-CATCCGTAAAGACCTCTATGCCAAC-3 and reverse 5-ATGGAGCCACCGATCCACA-3. Relative expression levels of ATGL and HSL to beta-actin were calculated as 2^–ΔΔCT^.

### Western blot analysis

The liver tissues were lysed in RIPA buffer and the protein concentration was determined using the BCA kit. Cell membrane or cytoplasmic extracts for the detection of p47phox (membrane) or FOXO1 (cytoplasm) were prepared by using the appropriate kits (Beyotime, China). Equal amounts of protein were separated by 10% SDS-PAGE and transferred to nitrocellulose membrane. The membranes were blocked in 5% non-fat milk in PBS and incubated with primary antibodies (1:1000 dilution) against related proteins overnight at 4°C, followed by incubation with HRP-labeled goat anti-rabbit secondary antibody (1:5000) at room temperature for 1 h. The immune complex was detected with an enhanced chemiluminescence system, were exposed to X-ray film, and analyzed. GAPDH was used as the loading control.

### Statistical analysis

Data are expressed as the mean ± S.D. Statistical analysis was performed using SPSS 11.0 statistical software. Differences among groups were compared by means of one-way ANOVA followed by the LSD test to determined significance in groups. *P* values <0.05 were considered statistically significant.

## Results

### Res supplementation reduces the body weight and liver index

**Figure 1:**
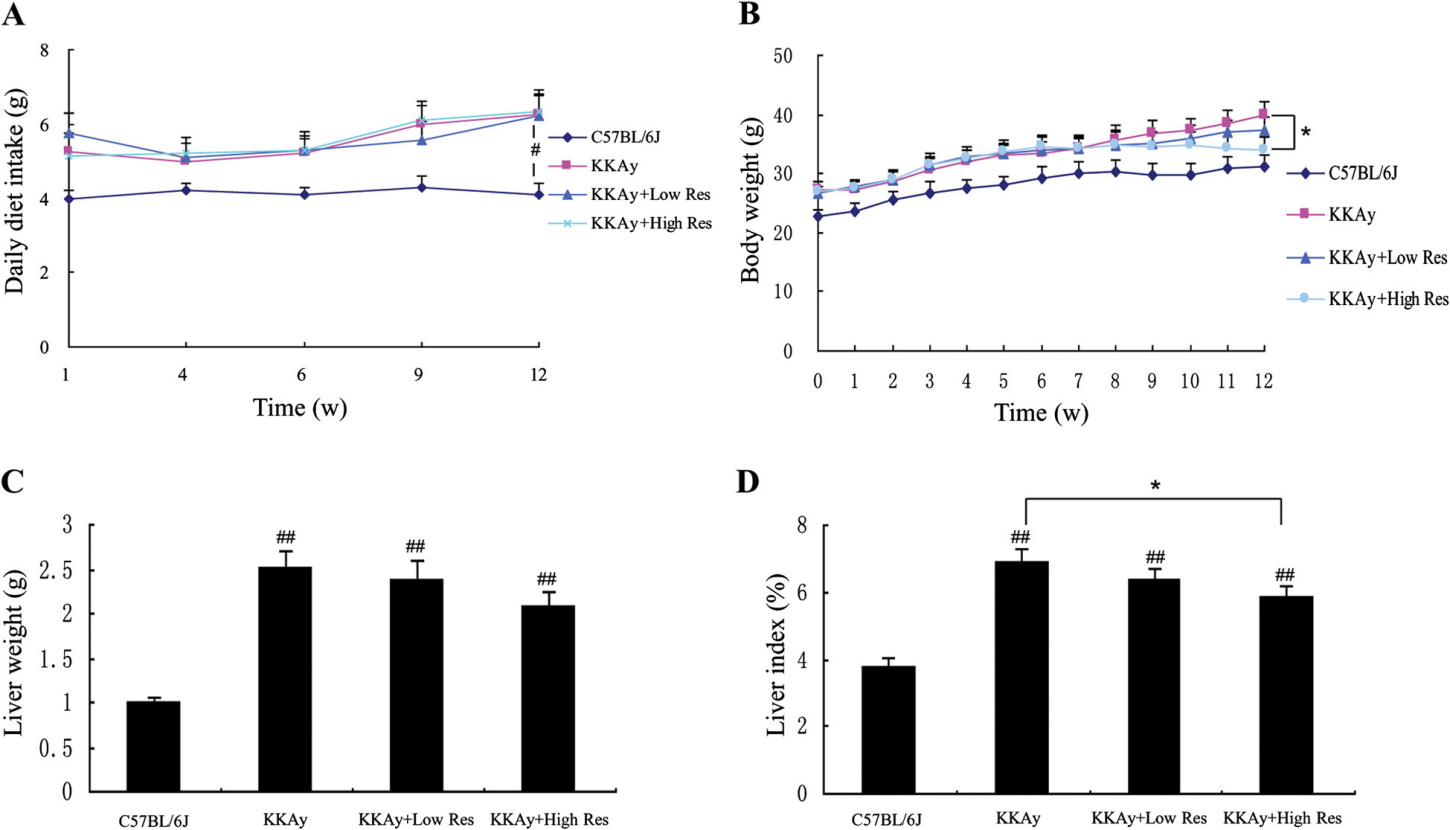
**Effect of dietary Res treatment on animal characteristics. (A)** Average daily food intake of each mouse at 1, 4, 6, 9, and 12 weeks. **(B)** Body weight (BW) was measured weekly throughout the study. **(C)** After the Res intervention, mice were sacrificed under anesthesia and the liver tissues were removed and weighed. **(D)** The liver index (%), which is equal to the liver weight/body weight*100%. Data are shown as the mean ± S.D. ^#^*P* < 0.05 and ^##^*P* < 0.01 compared with C57BL/6 J mice. ^*^P < 0.05 vs. the control group of KKAy mice, *n* = 10.

During the experimental period, daily food intake of the KKAy mice was found to be constantly higher than that of C57BL/6 J mice (*P* < 0.05), while there was no significant difference among the three groups (Figure 1A). As shown in Figure 1B-D, body weight and liver index were higher in comparison to C57BL/6 J mice, but the change was reversed in the high Res treatment group.

### Dietary Res treatment regulates lipid metabolism and oxidative activation of KKAy mice

**Figure 2:**
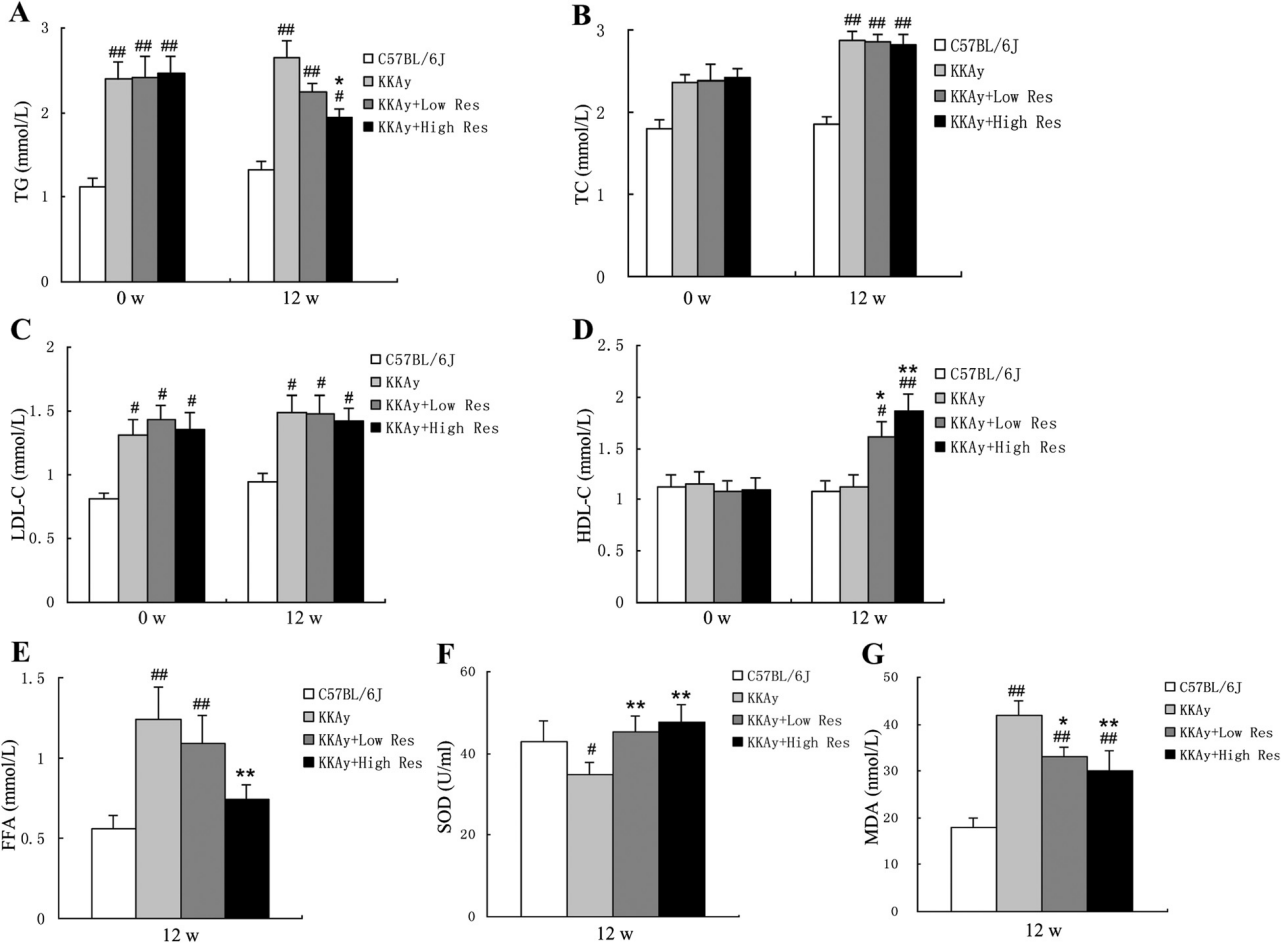
**Effects of dietary Res treatment on serum lipid levels and oxidative stress conditions. (A, B, C, D)** Serum levels of TG, TC, LDL-C and HDL-C, which represent serum lipid levels were measured before the Res treatment and after a 12-wk study period. **(E, F, G)**. Serum levels of FFA, SOD and MDA representing oxidative stress conditions were measured as well. Data are shown as the mean ± S.D. ^#^*P* < 0.05 and ^##^*P* < 0.01 vs. C57BL/6 J. ^*^P < 0.05 and ^**^P < 0.01 vs. KKAy control, *n* = 10.

As shown in Figure 2A-D, serum levels of TG, TC, and LDL-C in KKAy mice were higher than those in C57BL/6 J mice before and after 12-wk Res treatment (*P* < 0.05 or *P* < 0.01). Compared with non-treated KKAy mice, the serum level of TG was significantly decreased in the high Res treatment group (*P* < 0.01) and the HDL-C level was increased in both the high and low Res dietary groups (*P* < 0.05 or *P* < 0.01), but the TC and LDL-C levels were not significantly different. As shown in Figure 2E-G, serum levels of FFA and MDA in KKAy mice were higher than those in C57BL/6 J mice, while the SOD level was decreased (*P* < 0.05 or *P* < 0.01). In contrast to non-treated KKAy mice, FFA and MDA levels in the Res treatment group were significantly decreased, while the SOD level was increased (*P* < 0.05 or *P* < 0.01).

### Dietary Res treatment inhibits the liver oxidative stress

**Figure 3:**
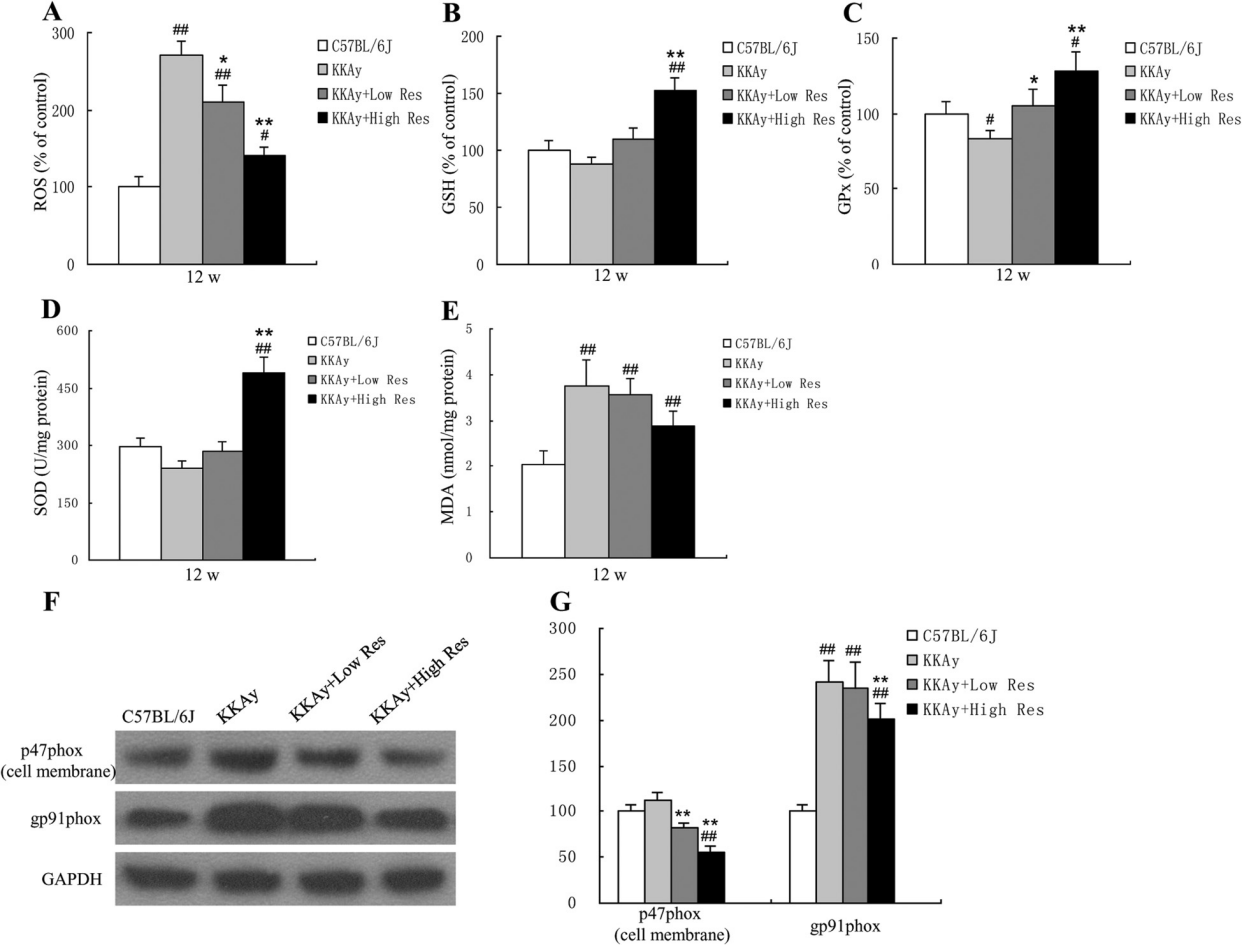
**Effects and mechanisms of dietary Res treatment on liver oxidative activation. (A, B, C, D, E)**. Hepatic levels of ROS, GSH, GPx, SOD and MDA were measured after a 12-wk study period. **(F)** Expression of p47*phox* (cell membrane), gp91*phox* and GAPDH in liver tissues in each group determined by Western blotting after intervention, each blot stands for at least three independent samples. **(G)** Quantitative analysis of expression levels, which were normalized for GAPDH. Data are shown as the mean ± S .D. ^#^*P* < 0.05 and ^##^*P* < 0.01 vs. C57BL/6 J. ^*^P < 0.05 and ^**^P < 0.01 vs. KKAy control, *n* = 10.

We explored the oxidative regulation effect of Res in liver tissues. As shown in Figure 3A-E, after the 12-wk study period, the ROS level was obviously decreased by dietary Res intervention. The MDA level also trended downward, but no significant difference was found. In addition, levels of glutathione (GSH), glutathione peroxidase (GPx), and SOD increased in our study (*P* < 0.05 or *P* < 0.01).

### Dietary treatment with Res decreases p47phox (cell membrane) and gp91phox expression

The protein levels of oxidative stress related proteins were detected by Western blot. As shown in Figure 3F and G, gp91phox protein levels were significantly increased in KKAy mice compared with C57BL/6 J (*P* < 0.01. Compared with non-treated KKAy mice, p47phox (cell membrane) and gp91phox protein were evidently decreased in KKAy mice treated with the Res diet (*P* < 0.01). The results suggest the anti-oxidation level of KKAy mouse liver tissue can be improved by Res treatment.

### Dietary Res treatment reduced hepatic steatosis

**Figure 4:**
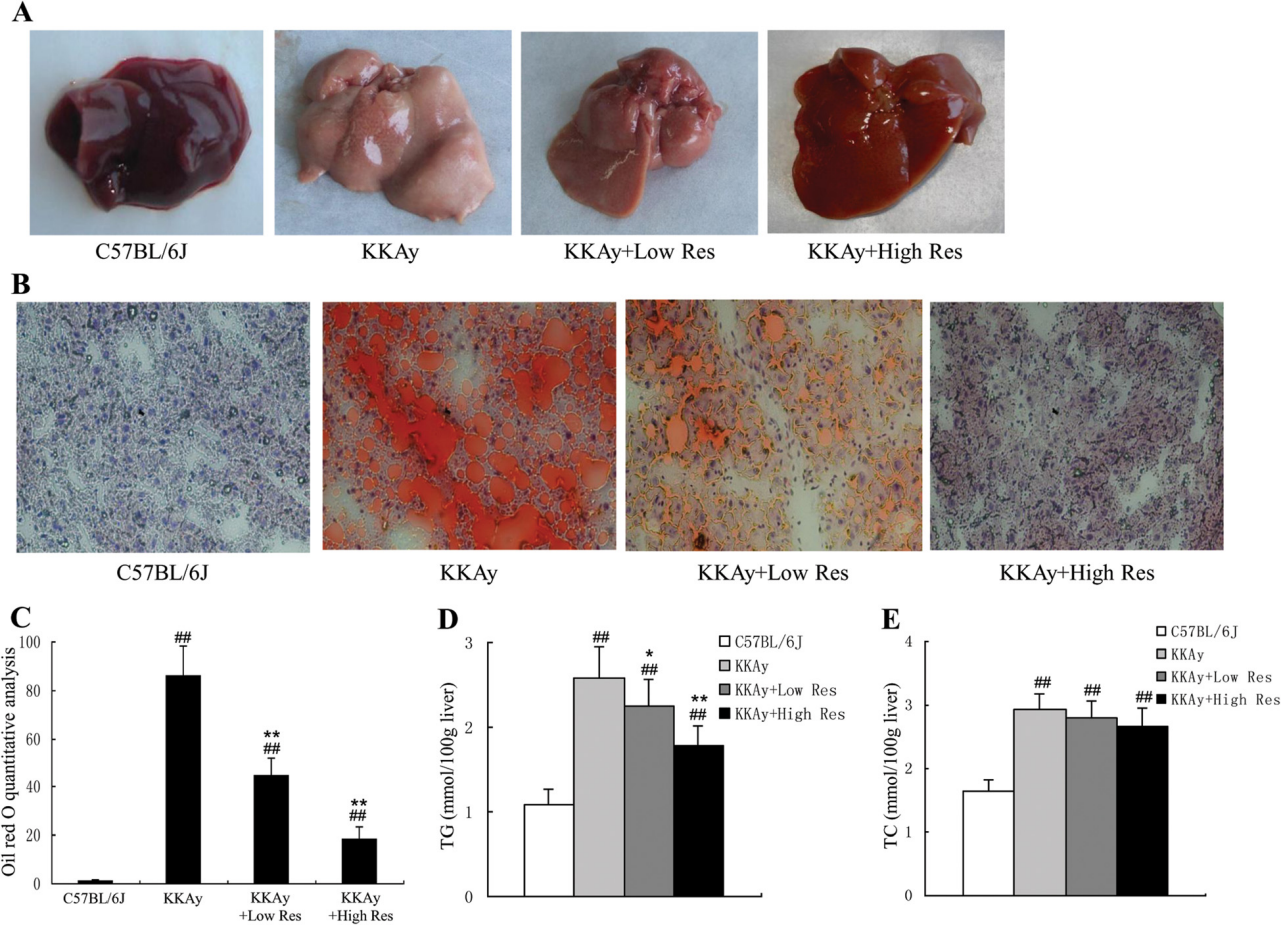
**Effects of dietary Res supplementation on hepatic steatosis in KKAy mice. (A)** Gross specimens of livers from mice in each group are shown. **(B)** Liver biopsy evaluation. Frozen sections of liver tissue were produced, the fat was stained with Oil Red O, and the nuclei were stained with hematoxylin. The original magnification was × 200. **(C)** The degree of hepatic steatosis of the mice in the different groups by Oil Red O quantitative analysis. **(D, E)** Hepatic levels of TG and TC in mice were measured. Data are shown as the mean ± S.D. ^##^*P* < 0.01vs. C57BL/6 J. ^*^P < 0.05 and ^**^P < 0.01 vs. KKAy control. *n* = 10.

We next examined the liver morphological alterations, the degree of hepatic steatosis, and liver TG and TC levels. Grossly, we found that Res treatment significantly attenuated the fat infiltration in the liver as shown in Figure 4A and B. Fresh liver tissue was bright red with smooth surfaces and sharp edges in C57BL/6 J mice, but in non-treated KKAy mice, the liver tissue was yellow and swelling, with granular surfaces and blunt edges. Examination of frozen sections of liver tissue demonstrated marked microvesicular steatosis and the degree of hepatic steatosis was significantly alleviated by the dietary intake of Res, as indicated by the reduced surface area of steatosis (Figure 4B). In parallel with the histological findings, Oil Red O quantitative analysis revealed that the fat deposits were markedly decreased by Res treatment (*P* < 0.01) (Figure 4C). Furthermore, as shown in Figure 4D and E, the liver TG level was reduced in Res-treated mice when compared with non-treated KKAy mice, whereas there was an insignificant change in TC level.

### Up-regulation of p-HSL, HSL and ATGL by dietary Res treatment in KKAy mice

**Figure 5:**
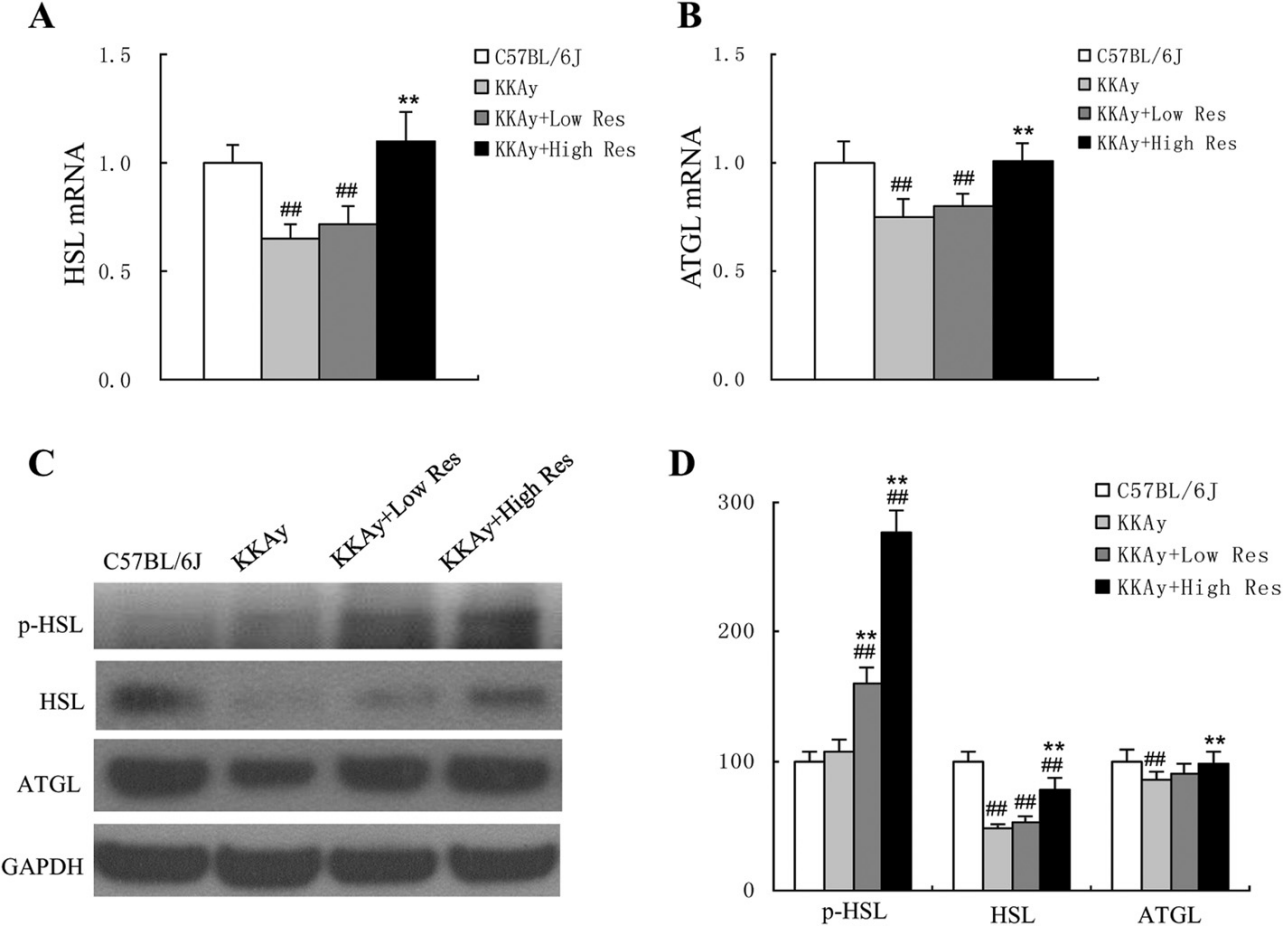
**Effects of dietary Res treatment on protein expressions of fat metabolism in liver tissues. (A, B)** HSL and ATGL mRNAs in liver tissues were measured by real-time PCR, and beta-actin mRNA was used as referred gene. **(C)** Protein expression of p-HSL, HSL and ATGL in liver tissues in each group were determined by Western blotting, each blot stands for at least three independent samples. **(D)** Quantitative analysis of protein expression levels, which was normalized for GAPDH. Data are shown as the mean ± S.D. ^##^*P* < 0.01 vs. C57BL/6 J. ^**^P < 0.01 vs. KKAy control, *n* = 10.

Real-time PCR was carried out to detect the mRNA levels of HSL and ATGL after a 12-w study period. As shown in Figure 5A and B, HSL and ATGL mRNA levels were significantly decreased in KKAy mice compared with C57BL/6 J, and this decrease was evidently reversed by high Res dietary treatment (*P* < 0.01). In agreement with PCR findings, Western blot analysis showed that ATGL and HSL proteins were up-regulated in KKAy mice treated with the high Res diet. Additionally, p-HSL protein was highly expressed in both the low and high Res treatment groups (*P* < 0.01), as shown in Figure 5C and D.

### Dietary treatment with Res increases Sirt1, p-AMPKα, p-FOXO1 and FOXO1 (cytoplasm) expression

**Figure 6:**
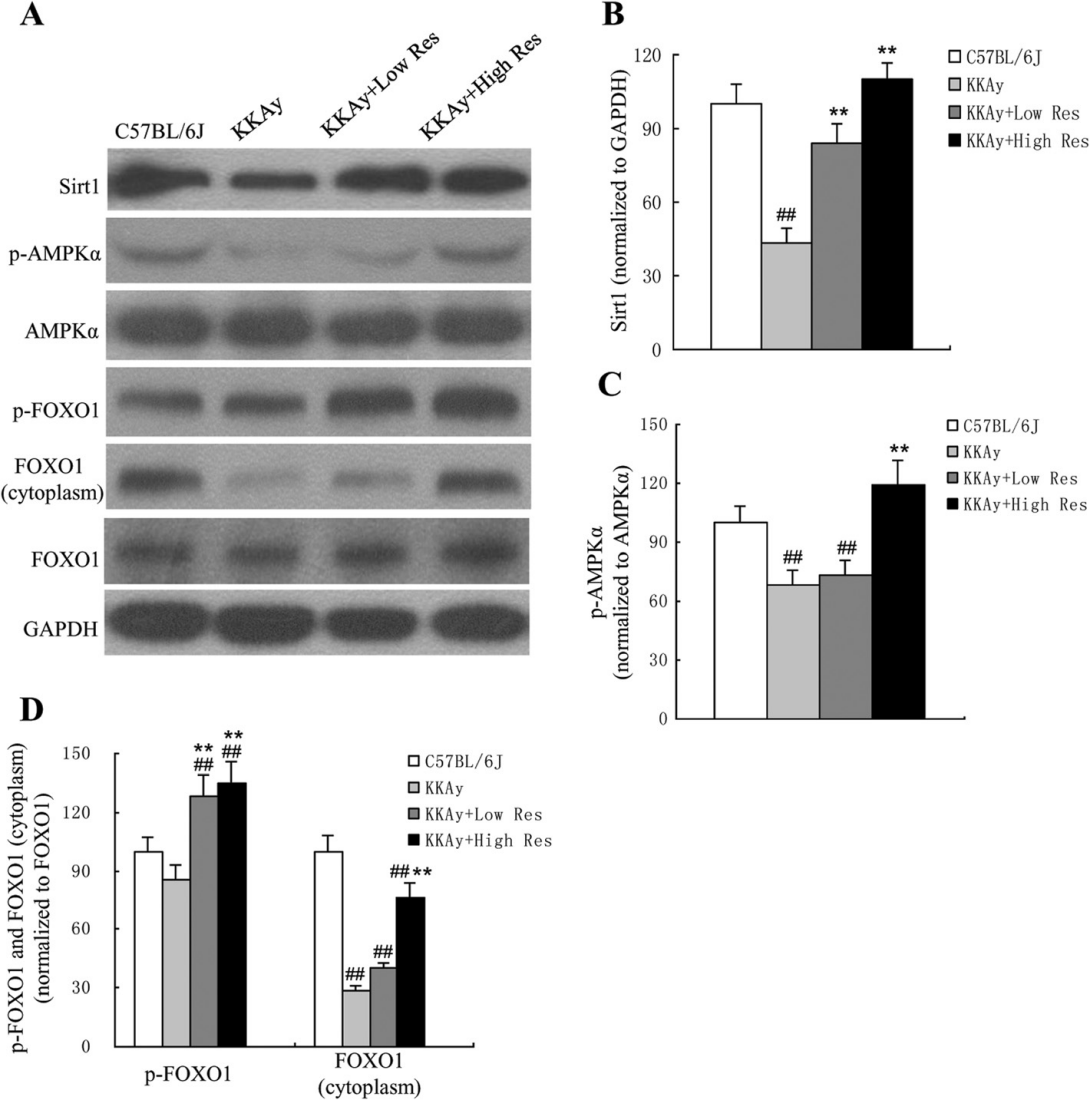
**Effects of dietary Res treatment on Sirt1, p-AMPK,AMPK, p-FOXO1, FOXO1 protein expressions in liver tissues. (A)** Sirt1, p-AMPK, AMPK, p-FOXO1, and FOXO1 protein expressions were measured by Western blotting, each blot stands for at least three independent samples. **(B, C, D)** Quantitative analysis of expression levels, which was normalized for GAPDH. Data are shown as the mean ± S.D. ^##^*P* < 0.01 vs. C57BL/6 J. ^**^P < 0.01 vs. KKAy control, *n* = 10.

Finally, we studied the possible underlying regulation mechanisms of Res. As shown in Figure 6, down-regulation of Sirt1, p-AMPK α, p-FOXO1 and FOXO1 (cytoplasm) in KKAy mice was reversed by Res treatment compared with C57BL/6 J mice. However, there was no effect on AMPK α or total FOXO1 protein expression.

## Discussion

Our study proves that Res can attenuate the effects in KKAy mice from oxidative stress and lipid metabolic disorders. Prior to Res intervention, KKAy mice present with weight gain and increases in TG, TC, and LDL-C levels compared to C57BL/6 J mice, moreover, after 12 weeks, non-treated KKAy mice developed severe oxidative stress and metabolic disorders in liver, leading to the unfavorable decreases of liver enzymes such as GPx, which is consistent with previous study supporting these mice as proper animal model for investigation of lipid metabolic disorders [18]. In our study, we investigated the effect of Res on lipid metabolism via dietary Res treatment. KKAy mice presented effective weight loss by Res dietary treatment, most likely due to inhibition of lipogenesis and/or lipid mobilization. Meanwhile, Res has anti-hyperlipidemic effects, as serum levels of TG were significantly decreased and HDL-C levels increased in the Res-treated groups, which may be the effect of improving the blood circulation and decreasing fat deposition via the inhibition of platelet aggregation [19]. In addition, Res-treated KKAy mice had decreased liver TG contents and lighter hepatosteatosis, accompanied by effective attenuation of oxidative stress in the liver. These anti-oxidation effects of Res were associated with higher GSH, GPx, and SOD levels in the livers of treated animals. Excessive oxidative stress plays an important role in the pathology of hepatic steatosis. Owing to its potent antioxidant effects, Res may be a potential therapeutic modality for NAFLD.

Generally, lipid and metabolic disorders are very likely to cause oxidant-antioxidant imbalances in the liver, where high levels of fatty acids provide the material basis for oxidative stress. Oxidative stress, due to the increased production of reactive oxygen species (ROS) and decreased antioxidant defense, was observed in both human and experimental models of steatohepatitis and appeared to be a key factor in the progression of NAFLD [20,21]. Excess levels of FFAs induced high levels of β-oxidation, and the production of ROS decreased antioxidant defenses at a mitochondrial respiratory chain level, simultaneously with the induction of necrosis [22]. Thus, the increased oxidative stress could lead to a greater risk for the development of NASH from simple steatosis. Expressions of p47phox and gp91phox have been found to increase in obese rats and be partly responsible for excessive oxidation [23]. Res appears to have a protective anti-oxidation effect through free radical scavenging [24]. Res has been found to be able to attenuate oxidative-induced DNA damage in human lymphocytes via increasing levels of GSH and modulating antioxidant enzymes (GPx, GR and GST) [25]. In our study, down-regulation of p47phox and gp91phox, increased production of GSH, GPx, SOD, and decreased serum MDA after Res treatment illustrate that Res has anti-oxidative effects, and thus ameliorates hepatocyte damage and steatosis. Moreover, we observed Res eventually reduced elevated serum FFA in KKAy mice, which may be due to the fact that Res intervention inhibited FFA synthesis, or enhanced FFA oxidation. Given that FFA level represents as important marker to evaluate metabolic disorder, the amelioration of Res on serum FFA pool would be of great significance.

As the hepatic steatosis is caused by increased lipogenesis and decreased lipolysis, we assumed that Res was able to reduce hepatic fat deposition via ATGL and HSL dependent lipolysis. HSL, the rate-limiting enzyme of intracellular TG hydrolysis, is a major determinant of fatty acid mobilization in adipose tissue as well as other tissues. In our research, HSL mRNA and protein levels were up-regulated in the Res-treated group. Furthermore, p-HSL protein was highly expressed in both the low and high Res-treated groups. These results show that Res treatment increases HSL expression at both the mRNA and protein levels, and enhances its lipolysis activity via phosphorylation. ATGL, an essential lipase in many cell types, specifically hydrolyses long-chain fatty acid TGs and plays an important role in lipolysis. Haemmerle G et al. reported that genetic inactivation of ATGL in mice increased adipose mass and led to triacylglycerol deposition in multiple tissues. ATGL-deficient mice accumulated large amounts of lipids in the liver, causing steatosis [26]. Atgl-/-hepatocytes exhibit defects in fatty acid metabolism and are steatotic [27]. Additionally, we have confirmed that ATGL expression was up-regulated after Res treatment in our study. Taken together, we showed that hepatic HSL and ATGL expressions were increased in Res-treated mice, and hepatic overexpression of HSL or ATGL reduces liver TG mass and ameliorates steatosis in KKAy mice. Based on previous reports and our findings, it is speculated that Res may promote lipolysis and ameliorate hepatic steatosis by activating HSL and ATGL.

Res has been reported to increase mitochondrial function by activating Sirt1 and extending the lifespan in multiple model organisms [28]. However, the exact mechanism of Res as direct Sirt1 activator has been challenged in previous studies. Beher *et al.*[29] reported that Res could increase Sirt1 activity in fluorophore-tagged substrates but not the corresponding nontagged peptides *in vitro*. More recently, Hubbard *et al.*[30] argued that sirtuin-activating compounds (STACs, resveratrol including) directly activate Sirt1 through an allosteric mechanism, moreover, the metabolic effects of STACs are blocked in cells reconstituted with inactive Sirt1, suggesting Sirt1 directly mediates STACs regulation *in vivo*. Despite of these inconsistent mechanistic investigations, Sirt1 was appeared to be involved in regulating energy homeostasis, food intake, body weight and substance metabolism [31]. Loss or reductions of Sirt1 activity may be associated with metabolic syndrome. Benefits of Sirt1 overexpression consisted of better glucose tolerance, as well as protection against hepatic steatosis [32]. Members of the FOXO family of transcription factors regulate the expression of numerous genes involved in the cell cycle, apoptosis, differentiation, development, DNA repair, and the cellular response to oxidative stress [33]. FOXO1 is regulated by post-translational modifications such as ubiquitination, phosphorylation, acetylation, etc. FoxO factors are considered to be downstream targets of the protein kinase Akt, which phosphorylates them and leaves them transcriptionally inactive by resulting in their nuclear exclusion and possibly subsequent proteasomal degradation [34]. However, the exact mechanism of the protective role of Res was largely unknown. In our study, increased expression of Sirt1 protein after the Res diet suggested that Res-induced Sirt1 activation may reduce steatosis, although mechanistically how Res activated Sirt1 remained unclear. Furthermore, down-regulation of Sirt1 and p-FOXO1 in KKAy mice were reversed by Res treatment, so we proposed that the Sirt1-FOXO1 pathway may be involved in resveratrol-mediated amelioration of oxidative stress. In detail, Sirt1 may increase Akt phosphorylation, which was reported to be activated after Res intervention in KKAy mice [15]. Akt promotes translocation of FOXO1 from the nucleus to the cytoplasm, consequently, liver gluconeogenesis was inhibited, and lipid metabolic disorder and oxidative activation were attenuated.

AMP-activated protein kinase (AMPK), an evolutionarily conserved serine/threonine protein kinase, is a heterotrimeric complex comprised of a catalytic subunit and two regulatory subunits. The primary mechanism responsible for AMPK activation involves phosphorylation of AMPK at the Thr172 residue located within the activation loop of the alpha subunit [35]. HMG CoA reductase and Acetyl CoA carboxylase (ACC) are the key enzymes in cholesterol and fatty acid synthesis, respectively, also well as being characterized as downstream targets of AMPK. Studies have determined that the activation of AMPK could inhibit cholesterol and fatty acid synthesis via phosphorylation of the two enzymes [36]. LKB1 is a serine-threonine protein kinase that phosphorylates and activates AMPK. Sirt1 affects downstream targets of LKB1. Thus, its overexpression increased AMPK and acetyl-CoA carboxylase phosphorylation, and conversely, RNA interference-mediated Sirt1 knockdown reduced AMPK phosphorylation [37]. However, recently Park *et al.*[38] reported that Res could elevate cAMP level and sequentially activate AMPK and Sirt1, indicating AMPK could be the upstream of Sirt1. Our results have revealed that Res may reduce steatosis via enhanced Sirt1 expression and increased AMPK phosphorylation. All of these findings suggest that there is correlation between Sirt1 and AMPK, but the exact regulating mechanism is uncertain.

In conclusion, we suggest that Res may attenuate fat deposition and ameliorate oxidative stress in a KKAy mouse model, most likely via up-regulation of Sirt1 and AMPK. Due to its protective role against hepatic steatosis, Res may be applied for the treatment of NAFLD. Further studies are warranted to determine whether Res may be recommended as an effective strategy for preventing and/or treating NAFLD in humans.

## Competing interests

The authors declare that they have no competing interests.

## Authors’ contributions

Conceived and designed the experiments: WZ, SFC, XF. Performed the experiments: WZ, SFC, YSS, ZLZ, ZLL. Analyzed the data: SFC, ZLL, WXL. Contributed reagents/materials/analysis tools: WHL. Wrote the paper: SFC, XHZ, ZLL. All authors read and approved the final manuscript.
